# Effect of tracheotomy timing on patients receiving mechanical ventilation: A meta-analysis of randomized controlled trials

**DOI:** 10.1371/journal.pone.0307267

**Published:** 2024-07-23

**Authors:** Rongrong Han, Xiang Gao, Yongtao Gao, Jihong Zhang, Xiaoyan Ma, Haibo Wang, Zhixin Ji

**Affiliations:** 1 Department of Otolaryngology, Weifang People’s Hospital, Weifang, Shan dong Province, China; 2 Department of Critical Care Medicine, Weifang People’s Hospital, Weifang, Shan dong Province, China; 3 Urology Department I, Weifang Hospital of traditional Chinese Medicine, Weifang, Shan dong Province, China; Sapienza University of Rome: Universita degli Studi di Roma La Sapienza, ITALY

## Abstract

**Purpose:**

We assessed the effects of tracheostomy timing (early vs. late) on outcomes among adult patients receiving mechanical ventilation.

**Methods:**

PubMed, Embase, Web of Science and Cochrane Library were searched to identify relevant RCTs of tracheotomy timing on patients receiving mechanical ventilation. Two reviewers independently screened the literature, extracted data. Outcomes in patients with early tracheostomy and late tracheostomy groups were compared and analyzed. Meta-analysis was performed using Stata14.0 and RevMan 5.4 software. This study is registered with PROSPERO (CRD42022360319).

**Results:**

Twenty-one RCTs were included in this Meta-analysis. The Meta-analysis indicated that early tracheotomy could significantly shorten the duration of mechanical ventilation (MD: -2.77; 95% CI -5.10~ -0.44; *P* = 0.02) and the length of ICU stay (MD: -6.36; 95% CI -9.84~ -2.88; *P* = 0.0003), but it did not significantly alter the all-cause mortality (RR 0.86; 95% CI 0.73~1.00; *P* = 0.06), the incidence of pneumonia (RR 0.86; 95% CI 0.74~1.01; *P* = 0.06), and length of hospital stay (MD: -3.24; 95% CI -7.99~ 1.52; *P* = 0.18).

**Conclusion:**

In patients requiring mechanical ventilation, the tracheostomy performed at an earlier stage may shorten the duration of mechanical ventilation and the length of ICU stay but cannot significantly decrease the all-cause mortality and incidence of pneumonia.

## Introduction

Many patients are admitted to the intensive care units (ICU) each year because they require mechanical ventilation [1]. Mechanical ventilation (MV) is usually performed through orotracheal intubation (OTI) or a tracheostomy tube. Tracheostomy is often considered when a patient requires prolonged mechanical ventilation or improved respiratory status [2]. Tracheostomy is one of the routine procedures for the critical care population. Tracheotomy can have a positive impact on the improvement of the patient’s respiratory function compared to continuous translaryngeal endotracheal intubation. Tracheotomy reduces injuries to the larynx and upper airway from endotracheal tube, facilitates the removal of secretions, increases the mobility of patients, reduces the need for sedation, and enhances the comfort of patients [2–5]. However, as an invasive operation, tracheotomy may lead to complications such as tracheal stenosis, incisional infection, hemorrhage, and fistula formation [6–8].

In recent years, many studies have explored the optimal timing of tracheotomy. However, the conclusions of many relevant studies are inconsistent [9–14]. Similarly, previous meta-analysis assessing the effects of early and late tracheotomy on patients undergoing mechanical ventilation have yielded different clinical outcomes [15–19]. The optimal timing of tracheotomy is a controversial subject and that is worth continuing to explore. Therefore, we performed this updated meta-analysis to determine the effect of tracheostomy timing on patients undergoing mechanical ventilation.

## Methods

### Search methods

Two investigators independently and systematically searched PubMed, Embase, Web of Science and Cochrane Library to identify RCTs about the early and late tracheotomy published up to September 2022. We used a combination of medical subject headings (MeSH) and keyword terms to search. The search terms included mechanical ventilation, ventilator, intratracheal intubation, tracheostomy, and tracheotomy. The search was limited to published articles. In addition, references mentioned in the identified articles were manually searched to identify additional studies that might be eligible.

### Selection criteria

The inclusion criteria: (1) Population: patients requiring mechanical ventilation in critical care units; (2) Intervention: early tracheotomy; (3): control: late tracheotomy; (4) outcomes: mortality, the incidence of pneumonia, mechanical ventilation days or ventilator-free days, length of hospital stay, length of stay in ICU. At least one outcome was reported. (5) study design: randomized controlled study.

The exclusion criteria: (1) non-RCT; (2) studies in children and neonates; (3) the required outcomes data were not reported; (4) without clearly defining the timing of tracheotomy; (5) duplicate articles.

### Data collection and study quality assessment

Two investigators independently screened studies based on the criteria, retrieved potentially relevant studies, extracted study data, and evaluated the quality of the eligible studies. Information extracted from these studies included the first authors, name, publication year, country, setting, sample size, patient characteristics (age, sex ratio, disease severity), inclusion and exclusion criteria, the timing of early and late tracheotomy, and major outcomes. Different studies have defined the timing of early and late tracheotomy differently. The primary outcome was mortality and incidence of ventilator-associated pneumonia. The secondary outcomes included duration of mechanical ventilation, length of ICU stay and length of hospital stay. The quality evaluation of the included RCTs was evaluated using the methods recommended by the Cochrane systematic review manual for assessing risk of bias. Any disagreements between the investigators were resolved by third investigators reviewing the original study or consulting the corresponding author.

### Statistical analysis

Continuous data were presented as mean differences (MDs) and 95% confidence intervals, whereas binary data were presented as Relative risk (RRs) and 95% confidence intervals. Heterogeneity of included studies was tested by chi-square test and quantitatively assessed using the I^2^ value. I^2^ < 50.0% or P > 0.1 was considered as no significant heterogeneity. If the heterogeneity between studies was not significant (p ≥ 0.1, I^2^ ≤ 50%), the fixed effects model was used for analysis. If there was significant heterogeneity among the included studies (p < 0.1, I^2^ > 50%), the random effects model was used for analysis. Publication bias was evaluated using the funnel plots. Subgroup analysis of mortality and ventilator-associated pneumonia was performed according to different study characteristics. These data were analyzed using RevMan 5.4 system software and Stata 14.0 software. The robustness of the pooled results was assessed by sequentially excluding individual studies. Significance is defined as 2-sided P <0.05.

## Results

### Eligible literature search results and study characteristics

**Fig 1:**
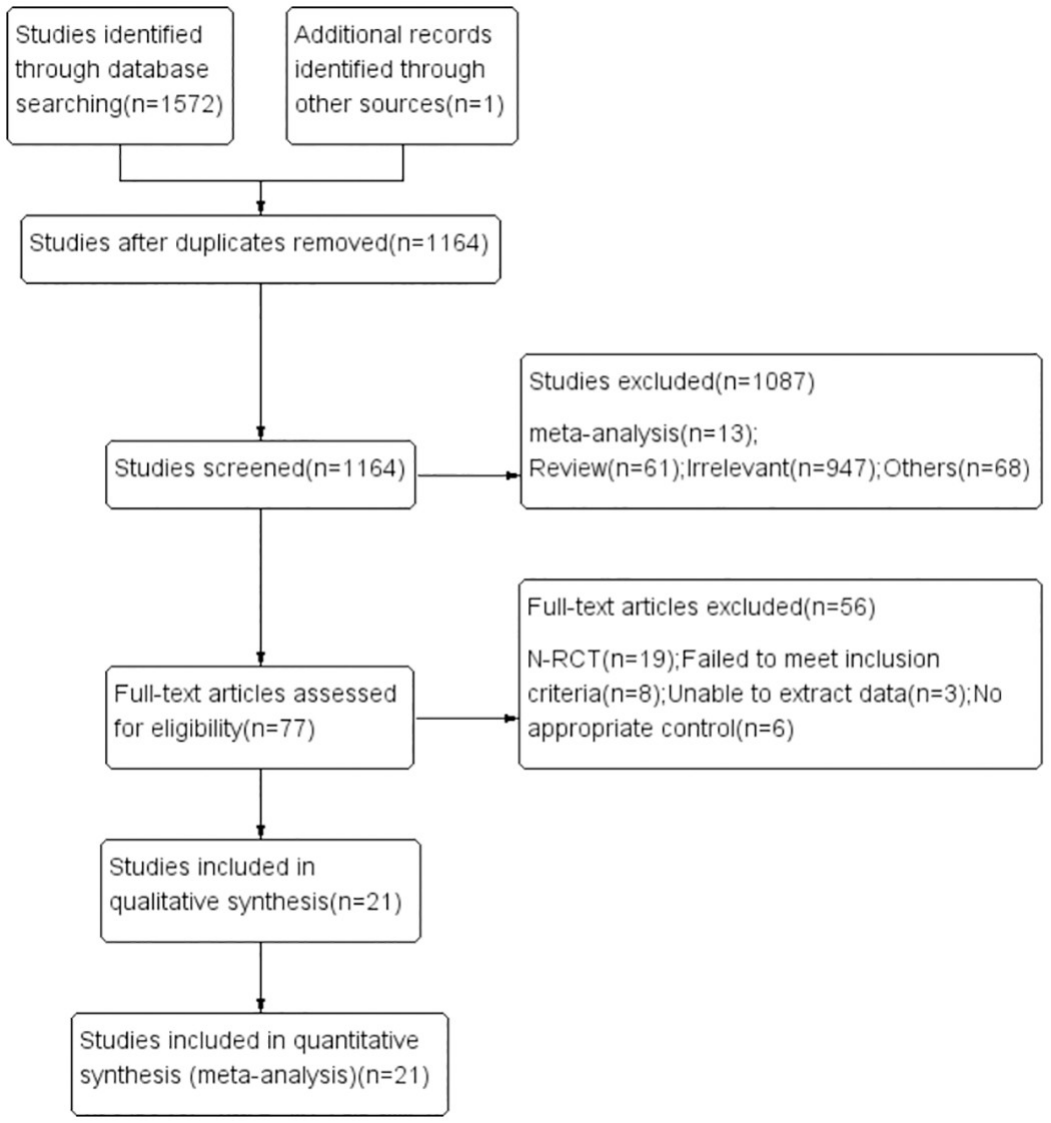
Study inclusion flowchart.

We manually searched the references from similar studies and identified a new eligible study [28]. A total of 1573 potential articles were identified based on our search strategy. 409 articles were excluded due to duplication. Eventually, 21 RCTs [5, 10, 20–38] involving 3621 patients were enrolled for this meta-analysis. The flow diagram of the literature search and study selection is shown in Fig 1. There were 1796 in the early tracheotomy group and 1825 in the late tracheotomy group. Included patients had different indications for intubation, and the definitions of early and late tracheotomy varied in the trials. The baseline information of the eligible studies is shown in Tables 1 and 2.

**Table 1:** Baseline characteristics of studies included in the meta-analysis.

Study	Country	Setting	Sample size	Patient characteristics (E:early/L:late)		
			(E:early/L:late)	Age(yr)	Sex(M:men/W:women)	Severity scoring system
Rodriguez 1990 [20]	US	Trauma	106 E: 51/L: 55	E: 36±2 L: 39±2	E: (M: 41/W: 10) L: (M: 43/W: 12)	APACHE II E: 10±1 L: 10±1
Sugerman 1997 [21]	US	Mixed	112 E: 53/L: 59	NA	NA	AIII E: 66±3 L: 55±3
Saffle 2002 [22]	US	Burn	44 E: 21/L: 23	E: 44.5±4.3 L: 51.3±4.0	NA	NA
Bouderka 2004 [23]	Morocco	Head injury	62 E: 31/L: 31	E: 41.1±17.5 L: 40.0±19.0	E: (M: 18/W: 13) L: (M: 20/W: 11)	SAPS E: 5.4±1.5 L: 6.0±3.8
Rumbak 2004 [24]	US	Medical	120 E: 60/L: 60	E: 63±10.4 L: 63±9.3	E: (M: 31/W: 29) L: (M: 34/W: 26)	APACHE II E: 27.4±4.2 L: 26.3±2.6
Barquist 2006 [25]	US	Trauma	60 E: 29/L: 31	E: 53.7±21.5 L: 49.9±18.3	E: (M: 20/W: 9) L: (M: 26/W: 5)	ISS E: 27.4±4.2 L: 26.3±2.6
Blot 2008 [5]	France	Mixed	123 E: 61/L: 62	E: 55±51.1 L: 58±50.3	E: (M: 45/W: 16) L: (M: 43/W: 19)	SAPS II E: 50±90.4 L: 50±60
Terragni 2010 [26]	Italy	Mixed	419 E: 209/L: 210	E: 61.8±17.4 L: 61.3±16.8	E: (M: 138/W: 71) L: (M: 142/W: 68)	SAPS II E: 51.1±8.7 L: 49.7±8.6 SOFA E: 7.9±2.6 L: 7.6±2.9
Trouillet 2011 [27]	France	After cardiac surgery	216 E: 109/L: 107	E: 64.1±13.3 L: 66.0±12.4	E: (M: 77/W: 32) L: (M: 66/W: 41)	SAPS II E: 47.2±12.4 L: 45.8±11.4 SOFA E: 11.6±3.5 L: 10.9±3.6 GCS E: 10.9±3.3 L: 11.4±3.0
Bylappa 2011 [28]	India	Mixed	44 E: 22/L: 22	E: 33.05±16.25 L: 32.86±12.69	E: (M: 13/W: 9) L: (M: 17/W: 5)	NA
Zheng 2012 [29]	China	Medical	119 E: 58/L: 61	E: 67.5±14.7 L: 67.9±17.6	E: (M: 39/W: 19) L: (M: 35/W: 26)	APACHE II E: 19.57±2.33 L: 19.56±2.53 SOFA E: 7.40±1.52 L: 7.28±1.70
Koch 2012 [30]	Germany	Neurosurgery or neurotrauma	100 E: 50/L: 50	E: 62.5±26.8 L: 55.0±18.5	E: (M: 29/W: 21) L: (M: 35/W: 15)	APACHE II E: 21±14.1 L: 22±3.3
Bosel 2013 [31]	Germany	Neurological illness, neurosurgery, or neurotrauma	60 E: 30/L: 30	E: 61±12 L: 61±13	E: (M: 20/W: 10) L: (M: 20/W: 10)	GCS E: 9±3.0 L: 8±3.7 NIHSS E: 21±5.9 L: 20±16.3 APACHE II E: 17±4.4 L: 16±5.9 APS E: 12±3.0 L: 12±5.2
Young 2013 [10]	UK	Mixed	899 E: 451/L: 448	E: 63.6±13.7 L: 64.2±13.3	E: (M: 263/W: 188) L: (M: 264/W: 184)	APACHE II E: 19.6±6.5 L: 20.1±6.0
Dunham 2014 [32]	US	Traumatic brain injury	24 E: 15/L: 9	E: 33±13 L: 37±16	NA	GCS E: 4±2.5 L: 4±0.9 ISS E: 28±11 L: 35±9 Head AIS E: 4.7±0.6 L: 4.9±0.3
Diaz-prieto 2014 [33]	Spain	Mixed	489 E: 245/L: 244	E: 64±52.6 L: 65.5±51.1	E: (M: 170/W: 75) L: (M: 159/W: 85)	APACHE II E: 20±25.9 L: 19±25.2 SOFA E: 9±13.3 L: 9±14.1 ISS E: 29±42.2 L: 30±25.2
Filaire 2015 [34]	France	After lung resection	78 E: 39/L: 39	E: 63.5±8.2 L: 59.9±7.8	E: (M: 33/W: 6) L: (M: 31/W: 8)	%DLco: E: 65.2±14.3 L: 65.5±18.1 VO_2_max (mL/kg/min): E: 19.5±3.9 L: 19.4±2.8 %ppoFEV1: E: 41.5±5.4 L: 37.8±9.2
KARLOVIC 2018 [35]	Bosnia and Hercegovina	Surgical and trauma	80 E: 38/L: 42	E: 60±13 L: 61.5±28	E: (M: 30/W: 8) L: (M: 27/W: 15)	APACHE II E: 23.61±8.14 L: 22.36±7.32 SOFA E: 13.97±2.65 L: 14.80±3.17
Goo 2022 [36]	Malaysia	Neurosurgical	39 E: 20/L: 19	E: 45.1±16.6 L: 48.4±23.0	E: (M: 17/W: 3) L: (M: 14/W: 5)	GCS E: 7.7±4.3 L: 8.2±4.3
Bosel 2022 [37]	Germany and the US	Ischemic stroke, intracerebral hemorrhage, or subarachnoid hemorrhage	380 E: 186/L: 194	E: 59.3±11.7 L: 57.6±12.0	E: (M: 90/W: 96) L: (M: 101/W: 93)	NIHSS E: 21±17.6 L: 21±17.6 GCS E: 7±3.7 L: 6±4.4
Eeg-Olofsson 2022 [38]	Sweden	COVID-19	61 E: 27/L: 34	E: 61.7±14.7 L: 65.0±9.0	E: (M: 20/W: 7) L: (M: 26/W: 8)	SAPS III E: 51.2±9.6 L: 50.2±5.3

Abbreviations: NA, not acquire; APACHE II, acute physiology and chronic health evaluation II; AIII, chronic health evaluation III; SAPS, simplified acute physiology score; ISS, injury severity score; SOFA, sequential organ failure assessment; GCS, Glasgow Coma Scale; APS, Acute Physiology Score; AIS, Abbreviated Injury Score; NIHSS, National Institutes of Health Stroke Scale; %DLco, percentage of diffusing capacity for carbon monoxide by single breath; %FEV1, percentage of forced expiratory volume in 1 second; FiO2, fraction of inspired oxygen; VO2max, maximal oxygen consumption; ppo, predictive postoperative; COVID-19, coronavirus disease 2019.

**Table 2:** Inclusion and exclusion criteria for the study, time of tracheotomy of included studies.

Study	Criteria		Early tracheotomy	Late tracheotomy	Tracheotomy methods
	Inclusion	Exclusion			
Rodriguez 1990 [20]	Patients with multiple injuries requiring mechanical ventilation	Patients who did not require ventilator therapy >1 d; patients being actively disengaged from the ventilator; patients who died in the first 24 h.	Within 7 days of admission	later than 8 days	NA
Sugerman 1997 [21]	Intubated and required mechanical ventilation for 3 d; anticipated need for ventilatory support for ≥7 d	Age <18 years; patients with major burns or inhalation injury	3–5 days	14 days	Both
Saffle 2002 [22]	Age >18 years; hospitalized within 24 h of acute burn injury; ongoing mechanical ventilatory support on postburn day 2	Pregnant women; preexisting significant renal or hepatic disease; corticosteroid use before admission; patients who did not have cutaneous burn injuries	4 days	14 days	Open
Bouderka 2004 [23]	Isolated head injury with GCS ≤8 on admission; cerebral contusion on CT scan; GCS <8 on fifth day without any sedation	Not reported	on the 5th or 6th day after admission	prolonged endotracheal intubation	NA
Rumbak 2004 [24]	Age >18 years; projected to need ventilation >14 d; APACHE II score >25	Anatomical deformity of the neck; previous tracheotomy; platelet count <50 × 10^3^/μL, activated partial thromboplastin time/prothrombin time >1.5 times, or bleeding time >2 × normal; soft tissue infection of the neck; mechanical ventilation with a PEEP >12 cmH_2_O; intubated >48 h; neck on which it was technically difficult to perform a PDT	within 48h	at days 14–16	Percutaneous
Barquist 2006 [25]	older than 15 years of age, had been intubated at least 3 days when they were 7 days after admission to the Trauma ICU and had either a Glasgow Coma Score (GCS) >4 with a negative brain computed tomography (CT) (no anatomic headinjury), or a GCS >9 with a positive head CT (known anatomic head injury).	pre-existing tracheostomy or need for a tracheostomy as part of a scheduled surgical procedure, contraindication to surgical tracheostomy and patients without a trauma related diagnosis.	before day 8	after day 28	Open
Blot 2008 [5]	Age >18 years; expected intubation >7 d	Previous tracheotomy or enrollment in the trial; major risk of bleeding; infection or anatomical deformity of the neck; severe respiratory insufficiency or neurological failure; high severity of illness scores	within 4 days	after at least 14 days of MV	Open
Terragni 2010 [26]	Age >18 years; intubated for 24 h; SAPS II score 35–65; SOFA score ≥5; did not have a pulmonary infection (CPIS<6)	COPD; anatomical deformity of the neck; cervical tumors; history of esophageal, tracheal or pulmonary cancer; previous tracheotomy; soft tissue infection of the neck; hematologic malignancy; pregnant	after 6–8 days of laryngeal intubation	After 13–15 days of laryngeal intubation	Percutaneous
Trouillet 2011 [27]	Undergone cardiac surgery; still mechanical ventilation 4 d after surgery; unsuccessful mechanical ventilation screening test result or spontaneous breathing trial on the day of randomization; expected to require mechanical ventilation for ≥7 more d.	Age <18 years; pregnant; previously enrolled in this or other trials of morbidity or mortality; received >48 h of mechanical ventilation preoperatively; previous tracheostomy within 6 mo; received an artificial heart device; prothrombin time >1.5 × upper limit of normal; platelet count <50 × 10^3^/μL; irreversible neurological disorder; SAPS>80; decided to limit care; soft-tissue neck infections or anatomical deformities or concomitant neck or carotid surgery	before the end of calendar day 5 after surgery	prolonged intubation	Percutaneous
Bylappa 2011 [28]	Prolonged intubation due to noncorrosive poisoning, snakebites, head injuries, and respiratory paralysis due to neurological disease	Trauma to the neck; previous neck surgery; tracheostomy; scar/keloid/previous radiotherapy in the neck; chemotherapy; fungating growth in neck; granulomatous disease; infection to neck	5–7 days	8–15 days	Open
Zheng 2012 [29]	Age >18 years; treated with mechanical ventilation via endotracheal intubation	Anatomical neck deformity; thyromegaly; cervical tumors; hematologic malignant neoplasm; previous tracheotomy; pregnant; weaned or died 48 h after mechanical ventilation onset	on day 3 of MV	on day 15 of MV	Percutaneous
Koch 2012 [30]	Age >18 years; expected time of ventilation >21 d (decided by 2 independent intensivists not involved in the study)	Anatomical variants or deformities of the larynx/trachea; preexisting tracheostomy; preexisting pneumonia; critical trauma of the cervical vertebral column; coagulopathy (thrombocyte level<60×10^3^/μL; prothrombin time >40 s; INR>1.4); estimated to die within the next 24 h; planned permanent tracheostomy; >3 d of ventilation before entry into the study.	≤4 days after intubation	≥6 days after intubation	Percutaneous
Bosel 2013 [31]	Age ≥18 years; admission to neuro-ICU; nontraumatic ICH/SAH/acute ischemic stroke; expected intubation ≥2 wk	Ventilation >3 d; severe chronic cardiopulmonary comorbidities; anatomical or clinical conditions jeopardizing PDT; expected to require a permanent ST; enrolled in other trials; life expectancy <3 wk; pregnant	within 3 days from intubation	between day 7 and 14 from intubation	Percutaneous
Young 2013 [10]	Mechanical ventilation in adult critical care units; identified by the treating clinician in the first 4 d after admission; likely to require ≥7 d of ventilatory support	Requiring an immediate, life-saving tracheotomy; tracheotomy contraindicated for anatomical or other reasons; respiratory failure due to chronic neurological disease	within 4 days of ICU admission	on day 10 or later	Both
Douham 2014 [32]	Age 18–65 years; blunt trauma with admission GCS≤8; ICH on brain CT scan	Cardiac arrest, near-brain death, preexisting coagulopathy, or severe obesity	post-injury day 3–5	post-injury day 10–14	NA
Diaz-prieto 2014 [33]	Age >18 years; intubated >48 h	Prior tracheotomy; included in another trial; technical difficulty in performing PDT	Before day 8	from day 14 onwards of MV	Percutaneous
Filaire 2015 [34]	Age 18–79 years; preoperative diagnosis of lung cancer or high suspicion of lung cancer; predicted postoperative DLco ≥30%; 30% ≤ ppoFEV_1_< 50%; ppoVO_2_max ≥10 mL/kg/min; surgical approach by lateral or Posterolateral thoracotomy	Pregnant; preoperative tracheotomy; vocal cord paralysis; phrenic nerve paralysis on the operated side; neuromuscular disorders; previous pharyngeal or laryngeal surgery; anatomical deformity of the neck; video-assisted thoracoscopic surgery; lung resection less important than planned at the inclusion (ppoFEV1≥50%).	after the closure of the thoracotomy		Open
KARLOVIC 2018 [35]	Age >18 years; patients in surgical and trauma units; intubated >48 h; expected duration of mechanical ventilation ≥14 d based on diagnosis; SOFA score >5, APACHE II scores >10, PaO_2_≤60 mmHg with FiO_2_ 0.5 and PEEP of at least 8 cmH_2_O	Previous tracheotomy; anatomical deformity of the neck; hematologic malignant neoplasms; respiratory infection within the first 48 h of mechanical ventilation	after 2–4 days of mechanical ventilation,	after 15 days of mechanical ventilation,	Percutaneous
Goo 2022 [36]	Patients admitted to the neurosurgical intensive care unit that likely requires prolonged mechanical ventilation were recruited.	Patients with no chance of survival, cardiac arrest on arrival, coagulopathy sufficient to contraindicate tracheostomy, patients who required immediate tracheostomy, and patients who were extubated before tracheostomy.	before 7 days of mechanical ventilation	after 7 days of mechanical ventilation	NA
Bosel 2022 [37]	Patients requiring invasive mechanical ventilation after acute, nontraumatic ischemic stroke, intracerebral hemorrhage, or subarachnoid hemorrhage were eligible if the stroke-related early tracheostomy score (SETscore) was more than 10.	a premorbid modified Rankin Scale score of more than 1, reflecting at least slight disability, duration of invasive mechanical ventilation for more than 4 days, clinical conditions either prohibiting early tracheostomy or mandating a surgical tracheostomy, pregnancy, life expectancy of fewer than 3 weeks due to a medical condition or an anticipated withdrawal of life-sustaining therapies, participation in any other interventional trial, or inability to obtain informed consent.	within 5 days of intubation	not before day 10	Both
Eeg-Olofsson 2022 [38]	Age≥18 years; Patients who were intubated due to real-time, reverse transcription polymerase chain reaction (RT—PCR)-verifed, SARS-CoV-2 infection with ARDS according to the Berlin defnition; Patients who were hospitalized at the Sahlgrenska University Hospital in Gothenburg or at two other county hospitals within the Region Västra Götaland of Sweden; Patients in which a need for MV for more than 14 days after intubation could not be ruled out.	Patients where a tracheotomy performed within 7 days after intubation could be life threatening due to a poor medical condition; Patients with an anatomical abnormality of the neck impeding the tracheotomy procedure; Patients with no informed consent.	≤7 days after intubation	≥10 days after intubation	Both

Abbreviations: NA, not acquire; GCS, Glasgow Coma Scale; APACHE II, acute physiology and chronic health evaluation II; PEEP, positive end-expiratory pressure; MV, mechanical ventilation; SAPS, simplified acute physiology score; SOFA, sequential organ failure assessment; CPIS, Clinical pulmonary infection score; PDT, percutaneous dilational tracheostomy; ST, surgical tracheostomy; COPD, chronic obstructive pulmonary disease; INR, International normalized ratio; ICH, intracerebral hemorrhage; SAH, subarachnoid hemorrhage; %DLco, percentage of diffusing capacity for carbon monoxide by single breath; FiO_2_, fraction of inspired oxygen; VO_2_max, maximal oxygen consumption; ppo, predictive postoperative.%FEV1, percentage of forced expiratory volume in 1 second. ARDS, Acute Respiratory Distress Syndrome.

### Risk of bias of enrolled trials

**Fig 2:**
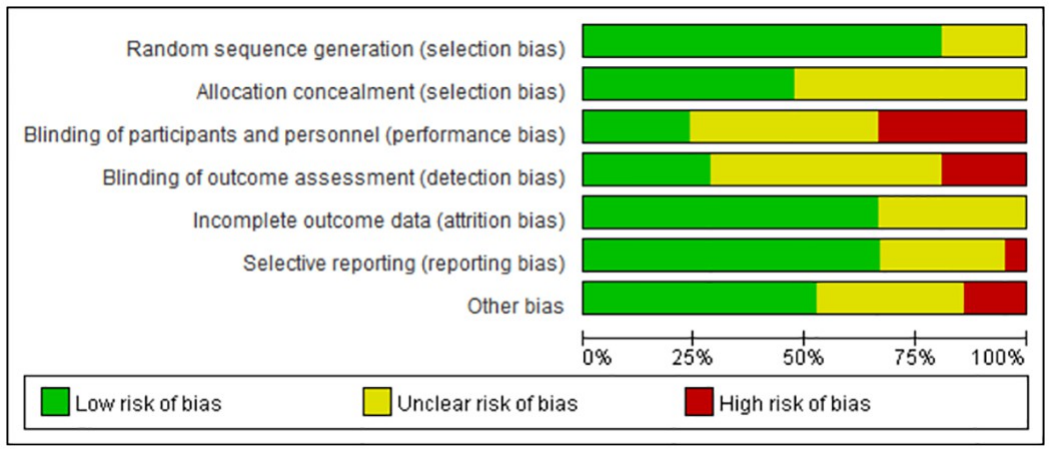
Quality evaluation of incorporated literature.

The quality evaluation revealed that the overall risk of bias of included trials was deemed to have low or unclear. Due to the characteristics of the intervention nature of tracheotomy, it was difficulty to blind clinicians and patients. However, objective outcomes, such as mortality, and incidence of ventilator-associated pneumonia, are unlikely to be affected by the absence of blinding. The quality evaluation is shown in Fig 2.

### Main results

#### Mortality

**Fig 3:**
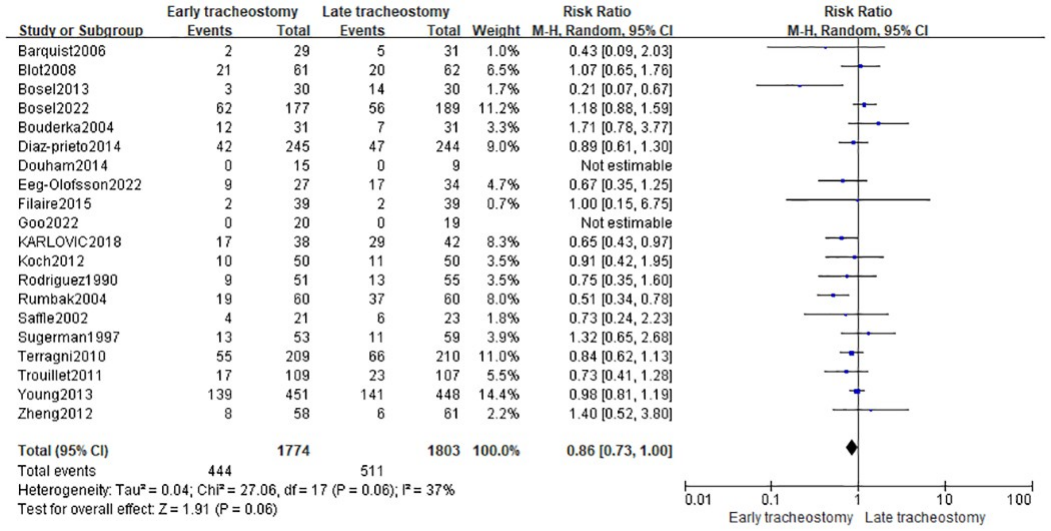
Effects of early versus late tracheotomy on the risk of mortality.

**Fig 4:**
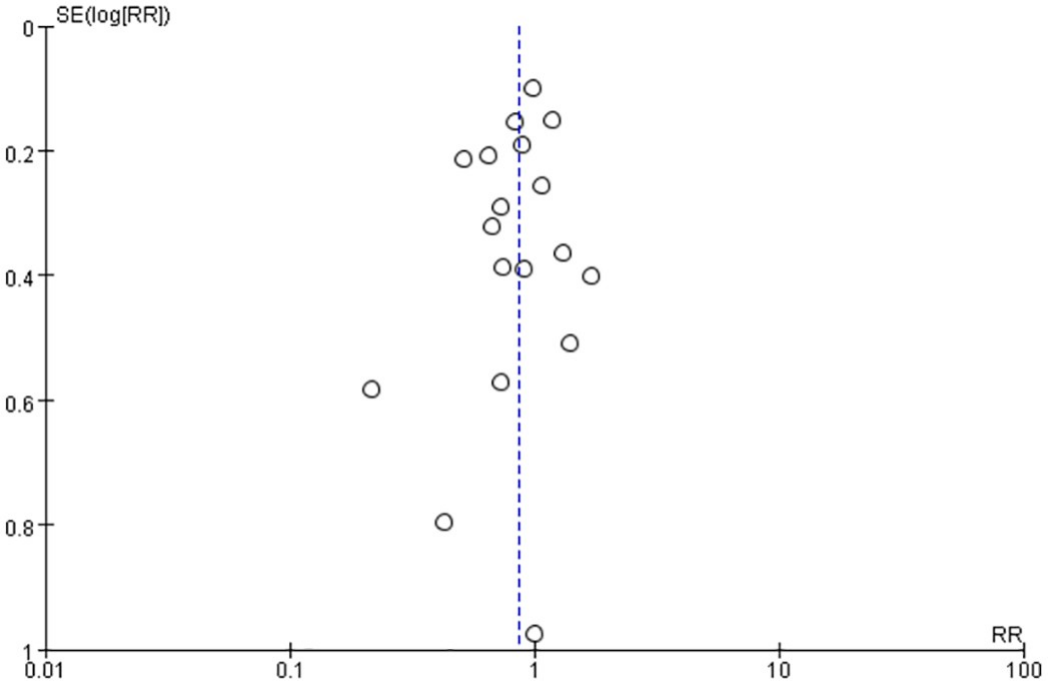
Publish bias funnel plots of mortality.

**Fig 5:**
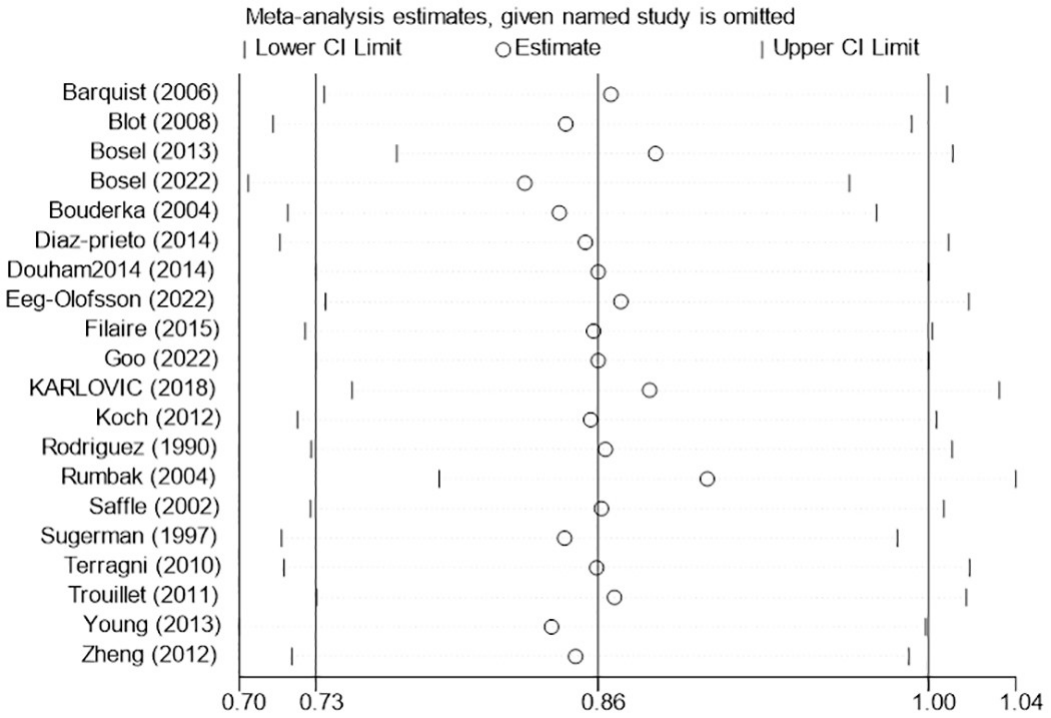
Sensitivity analysis of the effects of early versus late tracheotomy on the risk of mortality.

Twenty studies [5, 10, 20–27, 29–38] have reported mortality data with different timing of tracheotomy. The overall mortality in the early tracheotomy group was 25.03%, and in the late tracheotomy group was 28.34%. Because of the heterogeneity among the included studies was not significant (p = 0.06; I^2^ = 37%; Fig 3), the fixed model was used for the analysis. The analysis showed no significant difference in mortality between the early and late tracheotomy groups (RR 0.86; 95% CI 0.73–1.00; p = 0.06; Fig 3). There was no significant publication bias in funnel plots (Fig 4). The sensitivity analysis showed that this conclusion was not robust, and altered especially when the Bosel et al. study [37] was excluded (Fig 5). In addition, we performed subgroup analysis according to the different characteristics of the study. The subgroup analyses showed that early tracheotomy might have beneficial effects on the risk of mortality (mean age of patients ≥60.0 years, patients used percutaneous tracheotomy) (Table 3). The interaction *P* test showed that age (*P* = 0.03) might influence the treatment effect of tracheotomy timing on the mortality risk.

**Table 3:** Subgroup analyses for mortality and incidence of pneumonia.

Subgroup analyses for mortality						
Factors	subgroup	RR and 95%CI	*P* value	Heterogeneity (I^2^)	P-value of heterogeneity	P-value between subgroups
Country	US	0.68 (0.46–1.00)	0.05	25%	0.26	0.18
	Other	0.90 (0.77–1.06)	0.22	33%	0.12	
Sample size	≥100	0.91 (0.78–1.06)	0.23	28%	0.18	0.22
	<100	0.69 (0.45–1.06)	0.09	39%	0.13	
Etiology	Trauma	0.82 (0.50–1.34)	0.42	43%	0.15	0.89
	Neurologic	0.72 (0.31–1.64)	0.43	76%	0.01	
	Other	0.87 (0.75–1.00)	0.06	14%	0.31	
Mean age, y	≥60	0.76 (0.62–0.93)	0.07	45%	0.06	0.02
	<60	1.08 (0.87–1.34)	0.46	0%	0.59	
Percentage of male patients (%)	≥70	0.77 (0.60–0.98)	0.03	0%	0.76	0.47
	<70	0.87 (0.69–1.09)	0.23	62%	0.007	
Tracheotomy methods	Open	0.94 (0.61–1.44)	0.78	0%	0.69	0.06
	Percutaneous	0.72 (0.57–0.91)	0.005	38%	0.13	
	Both	1.02 (0.86–1.22)	0.79	11%	0.34	

#### Incidence of ventilator-associated pneumonia

**Fig 6:**
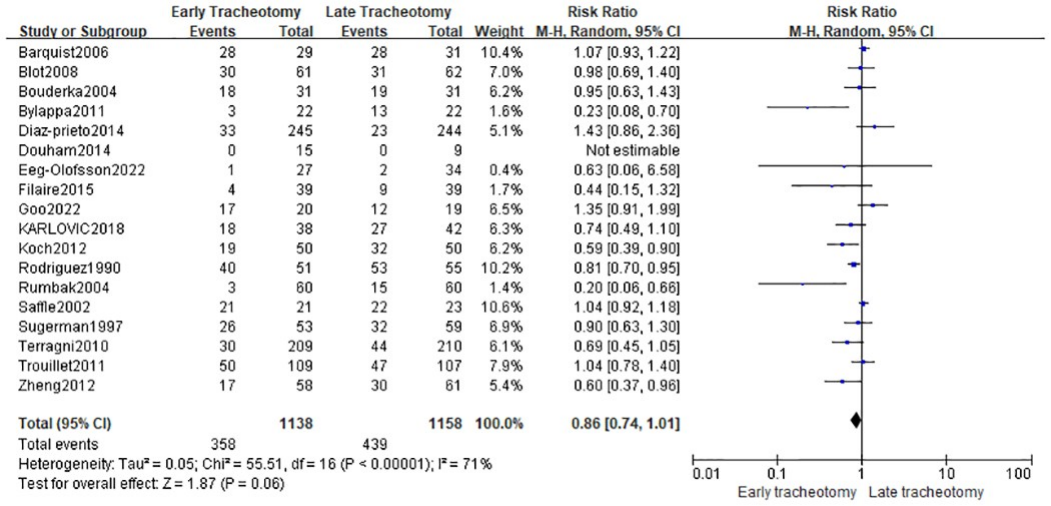
Effects of early versus late tracheotomy on the risk of pneumonia.

**Fig 7:**
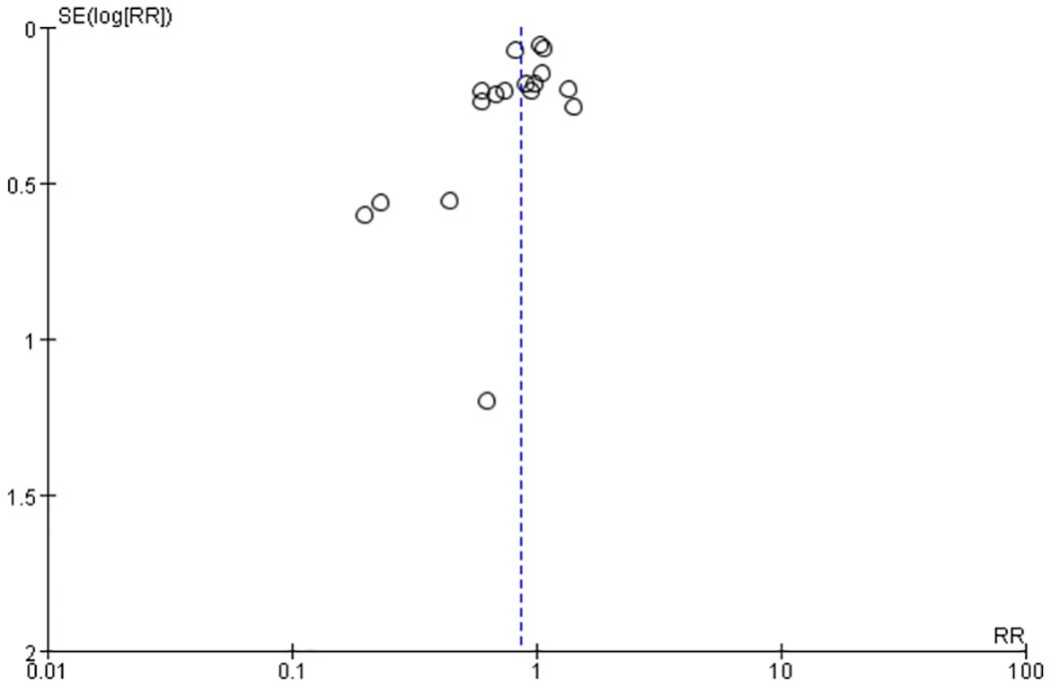
Publish bias funnel plots of pneumonia.

**Fig 8:**
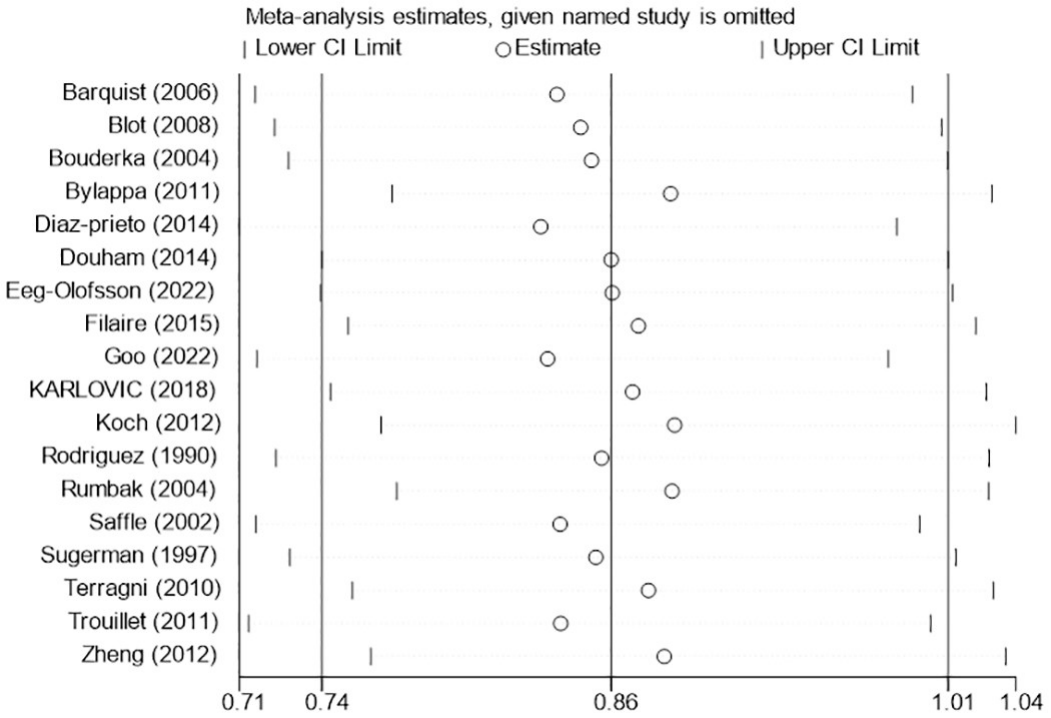
Sensitivity analysis of the effects of early versus late tracheotomy on the risk of pneumonia.

Eighteen studies [5, 20–30, 32–36, 38] reported data on the incidence of pneumonia. Pneumonia is a common clinical complication in mechanically ventilated patients. The overall incidence of pneumonia in the early tracheotomy group was 31.46%, and in the late tracheotomy group was 37.91%. There was significant heterogeneity among the included studies (I^2^ = 71%; P< 0.0001; Fig 6), so the randomized model was used for analysis. Although the incidence of pneumonia was lower in patients who underwent early tracheotomy, the difference was not statistically significant (RR: 0.86; 95% CI: 0.74–1.01; P = 0.06; Fig 6). There was no significant publication bias in funnel plots (Fig 7). The sensitivity analysis showed that this conclusion was stable and not changed by excluding any particular studies (Fig 8). The results of subgroup analysis showed that early tracheotomy was associated with a reduced risk of ventilator-associated pneumonia if the trial sample size was ≥100 (Table 4).

**Table 4:** Subgroup analyses for incidence of pneumonia.

Country	US	0.91 (0.71–1.15)	0.42	86%	<0.0001	0.61
	Other	0.83 (0.67–1.03)	0.09	57%	0.007	
Sample size	≥100	0.82 (0.68–0.99)	0.04	57%	0.02	0.47
	<100	0.92 (0.72–1.18)	0.51	76%	0.0001	
Etiology	Trauma	0.90 (0.73–1.11)	0.34	71%	0.02	0.79
	Neurologic	0.90 (0.39–2.08)	0.80	89%	0.003	
	Mixed	0.80 (0.63–1.04)	0.09	72%	0.0001	
Mean age, y	≥60	0.75 (0.55–1.03)	0.07	58%	0.02	0.28
	<60	0.92 (0.76–1.11)	0.39	79%	<0.0001	
Percentage of male patients (%)	≥70	0.88 (0.71–1.09)	0.24	69%	0.002	0.40
	<70	0.74 (0.51–1.06)	0.10	71%	0.002	
Tracheotomy methods	Open	0.90 (0.66–1.21)	0.48	83%	<0.0001	0.65
	Percutaneous	0.75 (0.56–1.01)	0.06	66%	0.007	
	Both	0.90 (0.63–1.30)	0.55	0%	0.76	

Abbreviations: US, the United States

### Secondary outcomes

#### Mechanical ventilation days or ventilator-free days

**Fig 9:**
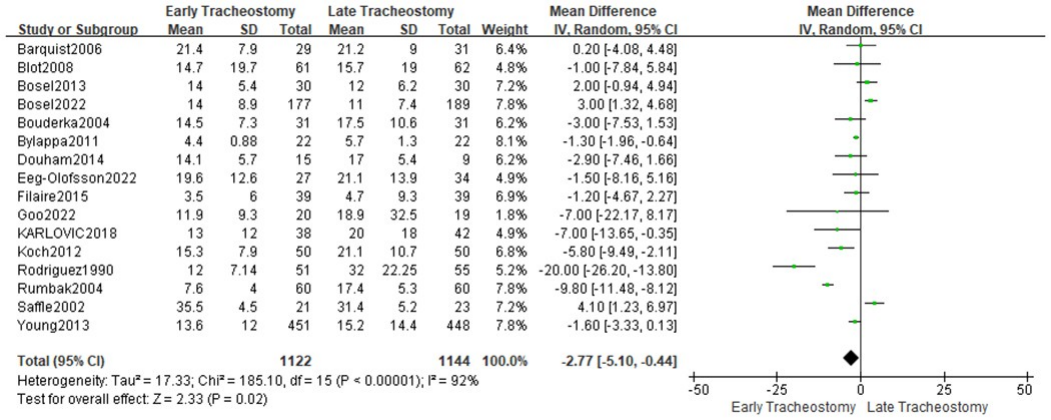
Effects of early versus late tracheotomy on the duration of mechanical ventilation.

**Fig 10:**
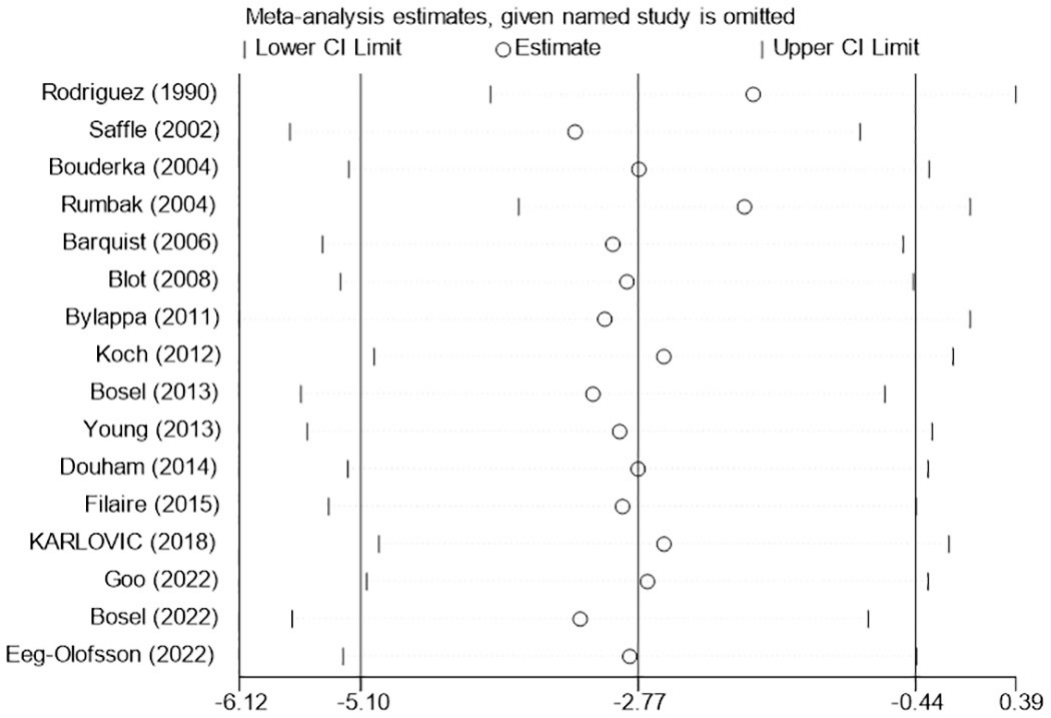
Sensitivity analysis of the effects of early versus late tracheotomy on the mechanical ventilation days.

**Fig 11:**
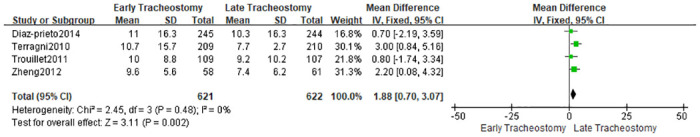
Effects of early versus late tracheotomy on the ventilator-free days.

Sixteen studies [5, 10, 20, 22–25, 28, 30–32, 34–38] reported data of mechanical ventilation days, and four studies [26, 27, 29, 33] reported data of ventilator-free days. Because of significant heterogeneity among the included studies (I^2^ = 92%; P< 0.0001; Fig 9), the randomized model was used for the analysis. The pooled analysis results showed that patients in the early tracheotomy group had shorter mechanical ventilation days (MD: -2.77; 95% CI: -5.10~-0.44; P = 0.02; Fig 9). However, sensitivity analysis (Fig 10) showed that this conclusion was not robust, especially after excluding the study of Rodriguez et al. [20]. The pooled analysis of ventilator-free days showed that more ventilator-free days for early tracheotomy patients (MD: 1.88; 95% CI: 0.70~3.07; P = 0.002; Fig 11), and the heterogeneity was not significant (I^2^ = 0%; P = 0.48). This is consistent with the above findings.

#### Length of ICU stay and length of hospital stay

**Fig 12:**
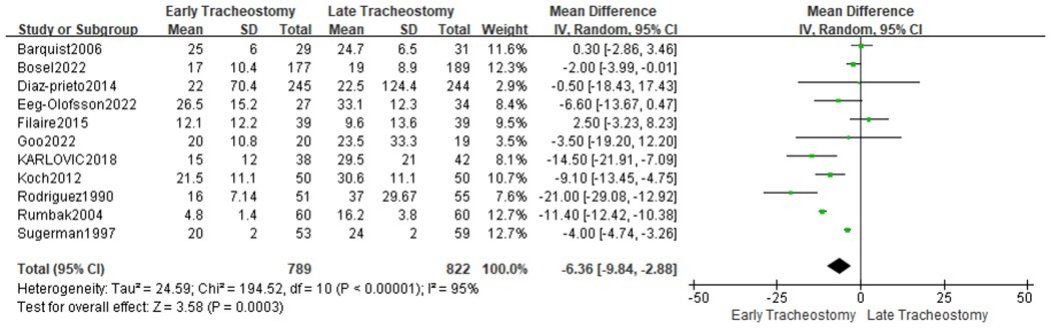
Effects of early versus late tracheotomy on ICU stay.

**Fig 13:**
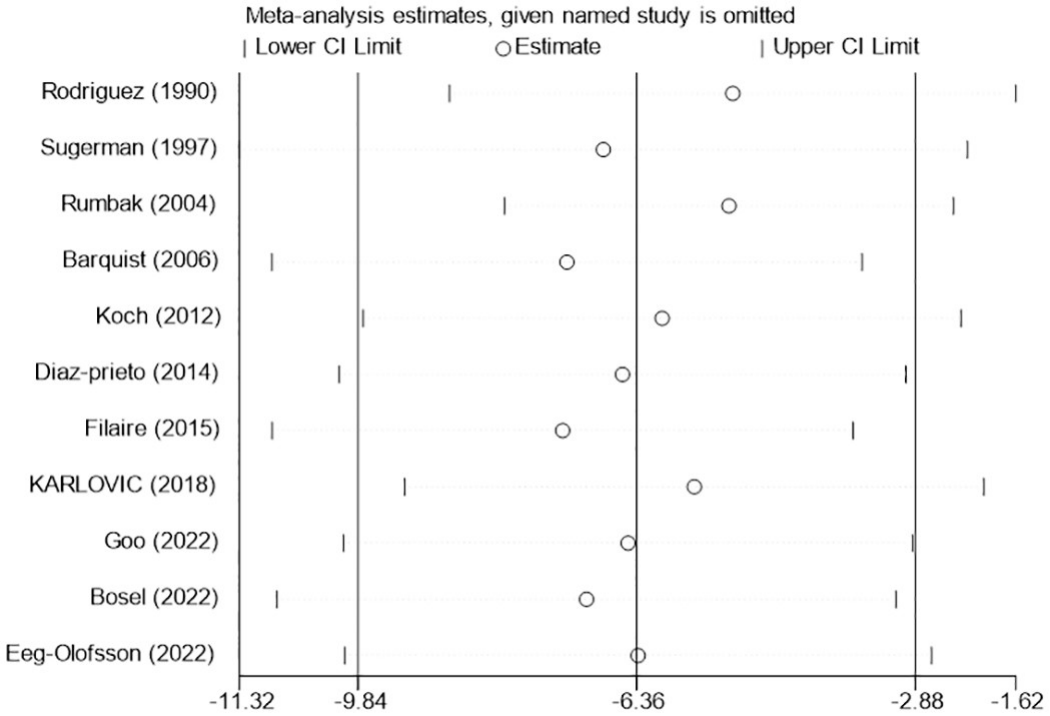
Sensitivity analysis of the effects of early versus late tracheotomy on the length of ICU stay.

**Fig 14:**
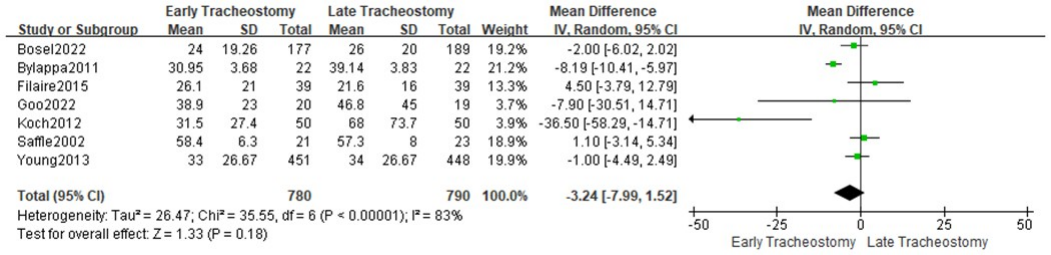
Effects of early versus late tracheotomy on hospital stay.

**Fig 15:**
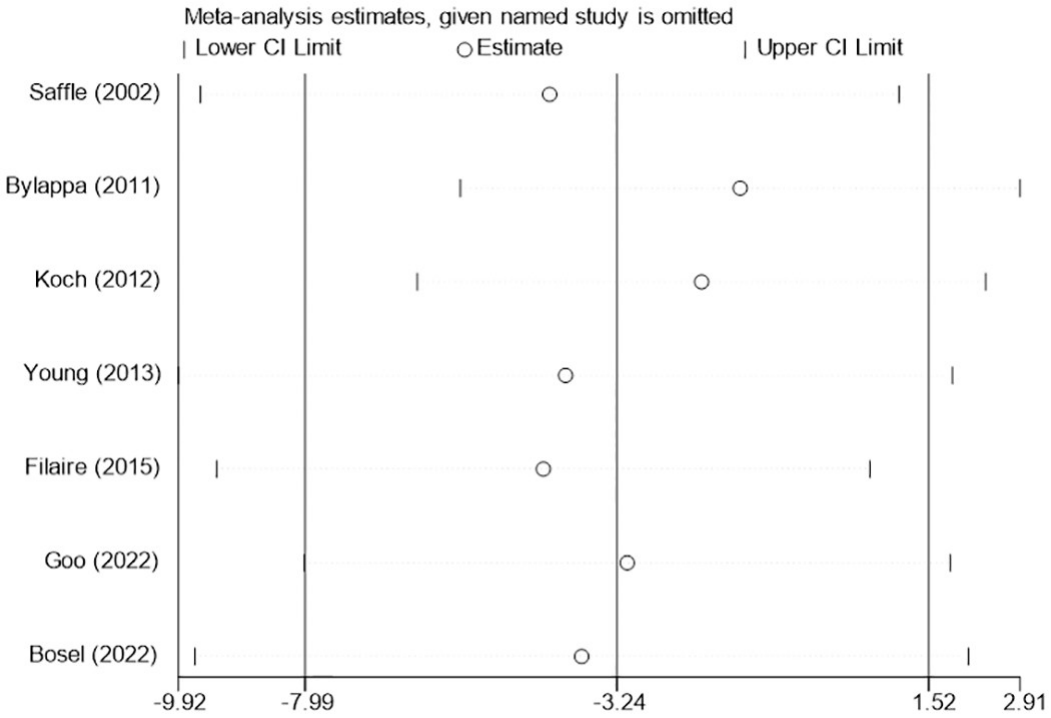
Sensitivity analysis of the effects of early versus late tracheotomy on the length of hospital stay.

Eleven studies [20, 21, 24, 25, 30, 33–38] provided data on the length of ICU stay. The analysis results showed that patients with early tracheotomy had shorter ICU stays (MD: -6.36; 95% CI: -9.84~-2.88; P = 0.0003; Fig 12), and there was significant heterogeneity among included studies (p<0.00001; I^2^ = 95%; Fig 12). The results of the sensitivity analysis showed that the conclusions were robust and not changed by excluding individual studies (Fig 13). Seven studies [10, 22, 28, 30, 34, 36, 37] provided data on the length of hospital stay. The results showed that the timing of tracheotomy was not associated with the length of hospital stay (MD: -3.24; 95% CI: -7.99~1.52; P = 0.18; Fig 14), and there was significant heterogeneity among included trials (p<0.00001; I^2^ = 83%; Fig 3H). This conclusion was not altered by excluding individual studies (Fig 15).

## Discussion

This updated meta-analysis evaluated the effect of tracheotomy timing on patients receiving mechanical ventilation. This study included a total of 21 randomized controlled trials (RCTs) involving 3621 patients. The included studies described in detail the inclusion and exclusion criteria for the observed population. In addition, most of the studies also comprehensively described the baseline data of the patients. The pooled analysis of this study showed that early tracheotomy reduced the duration of mechanical ventilation and length of ICU stay. Mortality and incidence of pneumonia were lower in the early tracheotomy group compared with the late tracheotomy group, but this was not statistically significantly different. In addition, we conducted subgroup analyses based on the characteristics of different studies to explore the effect of timing of tracheotomy on short-term clinical outcomes in mechanically ventilated patients. The subgroup analysis showed that early tracheotomy may have potential clinical benefits for patients aged ≥60 years or who underwent percutaneous tracheotomy. The clinical outcomes of early and late tracheotomy may be influenced by the age of the patients and the methods of tracheotomy.

In the ICU, many patients require mechanical ventilation. The coronavirus disease 2019 (COVID-19) pandemic in recent years has led to a significant increase in the number of patients requiring mechanical ventilation. Many patients positive for COVID-19 require prolonged mechanical ventilation in the ICU [39]. Tracheotomy is a commonly performed surgical procedure for patients who require prolonged mechanical ventilation. Nevertheless, the timing of tracheotomy in mechanically ventilated patients has been controversial. There are still no clear criteria to guide the timing of tracheotomy. A number of randomized controlled trials have explored the effects of early and late tracheotomy on mechanically ventilated patients. However, the conclusions were inconsistent. Similarly, some meta-analysis on this topic has reached inconsistent conclusions [15, 17, 19, 40]. The meta-analysis performed by Wang et al. [17] showed that early tracheotomy did not provide significant clinical benefits to patients requiring mechanical ventilation. However, a meta-analysis conducted by Chorath et al. [19] suggested that early tracheotomy was associated with a lower incidence of ventilator-associated pneumonia, shorter mechanical ventilation, and length of ICU stay. Novel RCTs [36–38] on this topic have recently been published. These studies were not included in the new meta-analysis. The inclusion of these newly published studies may address some of the questions on this topic. In addition, it should be pointed out that previous meta-analyses included many retrospective studies and did not perform stratified analyses.

To determine the impact of tracheotomy timing on patients, we included only randomized controlled trials in our meta-analysis and included newly published randomized controlled studies. We compared this study with recently published meta-analysis on this topic. Compared with the Chorath study [19], our study included seven other relevant RCTs [20, 21, 25, 32, 36–38]. Compared with the study of Deng et al. [41], we included seven additional relevant RCTs [28, 32, 34–38]. As far as we know, this is probably the meta-analysis to date involving the largest number of patients, and the results are more reliable.

The pooled results of the meta-analysis showed no statistical significance in mortality between the early and late tracheotomy groups. Sensitivity analysis showed that this result was not robust, and altered especially when the Bosel et al. Study [37] was excluded. This may be due to the higher proportion of young people in the study by Bosel et al. [37]. Our subgroup analysis showed that the effect of tracheotomy timing on mortality was influenced by the age of patients. Subgroup analysis found that early tracheotomy was associated with reduced mortality for patients underwent percutaneous tracheotomy. Percutaneous dilated tracheostomy offers the same safety as surgical tracheostomy [42]. Some studies [42–44] have shown that percutaneous tracheotomy reduces the overall incidence of inflammation and may further reduce clinically relevant hemorrhage rates and mortality. In addition, percutaneous tracheotomy does not require the involvement of the entire surgical team and the procedure takes less time. Percutaneous tracheotomy seems to be more clinically advantageous in the ICU [43, 44]. In the subgroup analysis of the effects of different etiologies on mortality, the 21 studies were divided into "trauma group", "neurology group" and "other disease group". However, subgroup analysis showed no reduction in mortality due to early tracheotomy in all three groups. This may be because we defined mortality as all-cause mortality in the study. Patients with tracheal intubation tend to have more complex conditions, and the complexity and severity of the disease can affect changes in all-cause mortality.

Pulmonary infections are a common complication in mechanically ventilated patients. Some studies [19] have indicated that early tracheotomy is beneficial to reduce the occurrence of ventilator-associated pneumonia. Nevertheless, this meta-analysis showed that early tracheotomy did not reduce the incidence of pneumonia. This is consistent with the results of Deng et al. [41]. The results of five [21, 25, 32, 36, 38] of the six RCTs [20, 21, 25, 32, 36, 38] newly included in this meta-analysis suggested that early tracheotomy was not associated with reduced incidence of pneumonia. It is not certain whether pneumonia is caused by ventilator ventilation or by other causes. It is important to note that tracheotomy itself can also lead to an increased incidence of pneumonia [45]. These may have some impact on the results. The possible reason is that tracheotomy disrupts the natural structure of the airway, reducing the protective effect of the airway and the cough reflex. In the ICU, the benefits of tracheotomy can be negated by frequent invasive procedures. In the subgroup study of the effects of different diseases on the incidence of pneumonia, early tracheotomy did not reduce the incidence of pneumonia in the "trauma group", "neurology group" and "other disease group". The incidence of pneumonia is not affected by the tracheotomy itself or its timing. The subgroup analysis showed that early tracheotomy might provide clinical benefit when the sample size of trials was ≥100.

Meta-analysis showed that early tracheotomy was associated with shorter mechanical ventilation days and length of ICU stay. Nevertheless, there was no significant difference in the length of hospital stay between the two groups. This may be because early tracheotomy was associated with reduced airway resistance, reduced respiratory work, reduced dead space, better secretion clearance, and enabling respiratory muscles to exercise and recover [41, 46]. Tracheotomy improves patients’ spontaneous breathing, thus reducing the duration of mechanical ventilation [47]. Moreover, compared with intubation, tracheotomy is more comfortable and reduces the need for analgesic and sedative drugs. This may also help patients regain respiratory function and reduce the length of mechanical ventilation and ICU stay. Sensitivity analysis showed that the conclusion of ventilator days was not stable, especially after excluding the study of Rodriguez et al. [20]. Rodriguez’s study included patients with multiple injuries requiring mechanical ventilation, which specifically included younger patients.

At present, there is no uniform standard for early and late tracheotomy timing. The definitions of the timing of early and late tracheotomy are also not uniform in different studies. In most studies, tracheotomy within 7 to 10 days after endotracheal intubation is considered as early tracheotomy. Therefore, the timing of early tracheotomy was defined as within 10 days after endotracheal intubation in this study. The primary condition for determining whether a patient needs an early tracheotomy is to accurately predict which patients are likely to require long-term mechanical ventilation. It is difficult to predict something accurately. If the prediction fails, a proportion of patients who do not require early tracheotomy may be included in the early tracheotomy group, thus affecting the reliability of the study results. This may lead to the misconception that patients in the early tracheotomy group are more likely to resume spontaneous breathing and have a lower incidence of ventilator-associated pneumonia. However, we have to admit that in clinical practice, there is still a lack of simple tools to accurately predict whether a patient needs long-term mechanical ventilation.

This meta-analysis also has some limitations. First, of the 21 RCTs included, only six [5, 10, 21, 26, 28, 33] were in comprehensive ICU patients, while the other studies were in specialized ICU patients. Second, there was significant heterogeneity in the included studies. The heterogeneity of this meta-analysis may be attributed to different inclusion and exclusion criteria for participants in different literature, different severity of disease in participants, different definitions of the timing of early and late tracheotomy, and different diagnostic criteria in different studies. For example, Barquist et al. [25] used the Centers for Disease Control and Prevention (CDC) diagnostic criteria for pneumonia, whereas Terragni et al. [26] used a simplified CPIS score (>6) to diagnose ventilator-associated pneumonia. Third, whether a patient requires long-term mechanical ventilation is often determined by the clinician based on the patient’s clinical manifestations, which can also influence the study results to some extent.

In conclusion, early tracheotomy reduces the duration of mechanical ventilation and length of ICU stay and does not significantly reduce the incidence of pneumonia, mortality, or length of hospital stay. However, there are many factors affecting the prognosis of tracheotomy, such as the severity of the disease, treatment. In addition, most of the patients in these studies were specialized ICU patients. Therefore, more high-quality and large sample RCTs involving comprehensive ICU patients are needed for further confirmation in order to obtain more reliable evidence-based medical evidence.

## Supporting information

S1 Checklist PRISMA 2020 checklist. (DOCX)
